# An NMR Metabolomic Study on the Effect of Alendronate in Ovariectomized Mice

**DOI:** 10.1371/journal.pone.0106559

**Published:** 2014-09-03

**Authors:** Shin-Yu Chen, Hui-Tzu Yu, Ju-Po Kao, Chung-Chun Yang, Shen-Shih Chiang, Darya O. Mishchuk, Jeng-Leun Mau, Carolyn M. Slupsky

**Affiliations:** 1 Department of Food Science and Biotechnology, National Chung Hsing University (NCHU), Taiwan, R.O.C.; 2 NCHU-UCD Plant and Food Biotechnology Center, NCHU, Taiwan, R.O.C.; 3 Agricultural Biotechnology Center, NCHU, Taiwan, R.O.C.; 4 Veterinary Medical Teaching Hospital, College of Veterinary Medicine, NCHU, Taiwan, R.O.C.; 5 Department of Food Science and Technology, University of California Davis, Davis, California, United States of America; 6 Department of Nutrition, University of California Davis, Davis, California, United States of America; Instituto de Investigación Sanitaria INCLIVA, Spain

## Abstract

Alendronate sodium (Fosamax) is most widely used for the prevention and treatment of osteoporosis. It is a type of anti-resorptive agent that reduces the risk of fractures by changing bone turnover and bone mineral density. We investigated the effect of Fosamax on a mouse model of osteoporosis. Twenty-seven female C57BL/6JNarl mice were divided into three groups: sham, ovariectomized (OVX) and OVX + Fosamax (Fosamax). After 23 weeks, bone density of femurs was analyzed using microcomputed tomography (micro-CT), and serum was analyzed for osteoblast and osteoclast activity, as well as metabolites using nuclear magnetic resonance (NMR) spectroscopy. Fosamax increased bone mineral density and cortical bone thickness, and decreased osteoblast activity slightly. Fosamax did not significantly change osteoclast activity. Serum metabolomics revealed that Fosamax had profound effects on overall metabolism, as significantly higher concentrations of metabolites associated with energy metabolism (including TCA-cycle intermediates and glucose), 3-hydroxybutyrate, taurine, allantoin, acetate, and ethanol, as well as lower concentrations of aspartate were observed in the Fosamax-treated mice compared with the OVX mice. These results suggest that alendronate may work by increasing bone density through altered metabolic activity.

## Introduction

Osteoporosis is a major public health issue. It is a skeletal disease that is defined by decreased bone mass combined with microarchitectural deterioration of bone tissue resulting in a consequent increase in bone fragility and susceptibility to fracture. Osteoporosis typically presents later in life, particularly in postmenopausal women, and its prevalence is expected to increase dramatically in the coming decades due to an ageing population [1], [2], [3]. The reason postmenopausal women are more susceptible to osteoporosis is due to reduced ovarian function resulting in decreased estrogen. Estrogen directly affects bone turnover by stimulating osteoblast activity (which forms bone) through increasing osteoblast formation, differentiation, proliferation, and function, and inhibiting osteoclast activity (which resorbs bone) through inducing osteoclast apoptosis, and inhibiting osteoclast formation [4], [5]. Thus, estrogen deficiency is directly related with bone loss, and postmenopausal estrogen deficiency causes accelerated bone loss. Postmenopausal osteoporosis affects 20% of women aged 60–69 years, and in the UK it was found that out of the 60,000 people who suffer osteoporotic hip fractures each year, 15–20% die from complications within a year [6].

Alendronate sodium (Fosamax), a nitrogen-containing bisphosphonate, is most widely used for the prevention and treatment of osteoporosis. Bisphosphonates accumulate in the mineral phase of bone and inhibit bone resorption through inhibition of osteoclast activity. The degree to which bone turnover and bone mineral density change upon treatment with anti-resorptive agents is directly correlated with a reduction in the risk of fractures [4], [7]–[10]. However, many side effects of bisphosphonate medications, including severe suppression of bone turnover that may develop during long-term therapy, actually increase the risk of fracture [11], [12]. Bisphosphonates can also cause osteonecrosis of the jaw (ONJ), with higher risk in oncology patients treated with high dose bisphosphonate therapy [13]–[15]. Other relevant possible side effects include gastrointestinal (GI) upset, musculoskeletal pain, atrial fibrillation, and esophageal cancer [16], [17].

There are few reports on the relationship between postmenopausal osteoporosis treatment with Fosamax and overall metabolism. To understand the global changes associated with Fosamax use, we investigated ovariectomized mice treated with Fosamax to determine its effects on serum metabolites using NMR spectroscopy. These results are compared with bone density using micro-CT as well as indicators of osteoblast and osteoclast activity using ELISA.

## Materials and Methods

### Ethics statement

This study was performed in strict accordance with animal use protocols approved by National Chung Hsing University Institutional Animal Care and Use Committee (IACUC, approval number: 99–62). All surgery was performed under anesthesia, and all efforts were made to minimize suffering.

### Animals and design

Twenty-seven female C57BL/6JNarl mice aged 7 weeks were purchased from the National Laboratory Animal Center (Taipei, Taiwan) and acclimated to conditions for 5 weeks before the start of the experiment. Animals were housed in an air-conditioned room in autoclavable cages (4–5 mice per cage) with ventilating tops and stainless steel lips (BioLASCO, Taipei, Taiwan) with 12 h light/dark illumination cycles at a constant temperature of 25±2°C and humidity of 65±5%. Tapvei aspen bedding was purchased from Young Li (New Taipei, Taiwan) and changed every week. Drinking water and food (basal diet Lab 5001 (Purina Mills, St. Louis, MO, USA)) were supplied *ad libitum*. The mice were weighed on a weekly basis during the experimental period, and food intake was measured from week 8 until the end of the experiment by subtracting the weight of the leftover food from the weight of the food provided on a weekly basis, and averaging across all mice.

Bilateral ovariectomy (OVX) was performed on 19 twelve weeks old mice after intramuscular injection of an anesthetic cocktail composed of 0.03 mL Zoletil 50 and 0.01 mL 0.2% Xylazine, as described by Lasota [18]. Another eight mice were given sham surgery. At 2 weeks post-surgery (week 0), 9 of the 19 ovariectomized mice were randomly assigned to the OVX (no treatment) group, and 10 to the Fosamax (treatment) group. The Fosamax group was then fed Fosamax plus (Merck, Whitehouse Station, NJ, USA) once a week based on the human equivalent dose [19], and contained 70 mg alendronic acid and 2800 IU of cholecalciferol. Blood was collected from the cheeks of mice after 12 weeks of treatment to measure osteocalcin and alkaline phosphatase (ALP). After 23 weeks, mice were sacrificed by CO_2_ asphyxiation, and blood was collected by cardiac puncture. Tissue was removed from each femur, and the bone was placed in ethanol until analysis. For serum, blood was allowed to clot for 15 minutes on ice, centrifuged at 5,000 rpm for 15 min, and the top layer was collected and stored at −80°C until ready for analysis.

### Histomorphometric analysis using microcomputed tomography

To perform qualitative and quantitative analysis of bone, the right femurs were analyzed using a high performance *in vivo* micro-CT Skyscan 1076 (Skyscan, Kontich, Belgium), with image field at pixel size 9 µm. Three-dimensional images were reconstructed using CTVol (Skyscan). The distal femoral metaphysis was analyzed from a region that was 1.0 mm below the growth plate and 1.5 mm in length. For quantitative analysis, the software CTAn (Skyscan) was used to obtain the following parameters within the region of interest (ROI): bone volume/tissue volume (BV/TV), bone surface/bone volume (BS/BV), bone surface/tissue volume (BS/TV), trabecular thickness (Tb.Th), trabecular separation (Tb.Sp), trabecular number (Tb.N), trabecular pattern factor (Tb.Pf), structure model index (SMI), connectivity density (Conn.Dn) and trabecular bone mineral density (Tb.BMD). The SMI quantifies the plate-rod characteristics of the 3D trabecular structure [20]. The Conn.Dn was calculated using the Euler method [21]. Cortical bone parameters (including cortical bone shell thickness, cortical BMD, endosteal circumference (EC), periosteal circumference (PC) and cross-sectional area) were analyzed in the middle of the diaphysis of the femur.

### Measurement of serum indicators of osteoblast and osteoclast activity, and serum concentrations of calcium and phosphate

We determined the activity of osteoblast and osteoclast cells using serum indicators rather than histomorphometry. Serum concentrations of alkaline phosphatase (ALP), osteocalcin, procollagen I C-terminal propeptide (PICP), pyridinoline (PYD), cross-linked N-telopeptide of type I collagen (NTXI) were all measured using commercial kits according to manufacturer's instructions. (ALP: Alkaline phosphatase activity fluorometric assay kit (Biovision, Mountain View, CA, USA); osteocalcin: Mouse osteocalcin EIA kit (Biomedical Technologies, Stoughton, MA, USA); PICP: ELISA kit for mouse PICP (USCN Life Science, Wuhan, Hubei, China); PYD: Mouse pyridinoline ELISA kit (CUSABIO, Wuhan, Hubei, China); NTXI: ELISA kit for mouse NTXI (USCN Life Science, Wuhan, Hubei, China)). Calcium was determined with a calcium colorimetric assay kit (Biovision, Mountain View, CA, USA), and phosphate was measured with a phosphate colorimetric assay kit (Biovision, Mountain View, CA, USA).

### Serum Metabolomics

For serum metabolomics measurement, serum samples were prepared by thawing and centrifuging at maximum speed for 20 min and 4°C to remove particulate matter. The serum supernatant was subsequently filtered through Amicon 3,000 molecular weight (MW) cutoff filters to remove protein and lipid particles. If needed, the serum filtrates were diluted with deionized water to a total volume of 207 µL and 23 µL of an internal standard (Chenomx Inc., Edmonton, Alberta, Canada) containing 4.8566 mM 3-(trimethylsilyl)-1-propanesulfonic acid-d 6 (DSS-d6) and 0.2% NaN_3_ in 98% D_2_O was added. The pH value was adjusted to 6.8 for each sample by adding small amounts of 1 N HCl or NaOH. 180 µL of each sample was transferred into a 3 mm NMR tube, and samples were stored at 4°C until NMR data acquisition (within 24 h of sample preparation).

NMR spectra were acquired using the Bruker noesypr1d experiment on a Bruker Avance 600 MHz NMR spectrometer equipped with a SampleJet as previously described [22]. Identification and quantification of metabolites were accomplished using Chenomx NMRSuite 7.6 (Chenomx Inc., Edmonton, Canada) [23]. For each mouse, 55 metabolites were measured and quantified from the serum which included 2-hydroxybutyrate, 2-oxoglutarate, 2-oxoisocaproate, 3-hydroxybutyrate, acetate, acetoacetate, acetone, alanine, allantoin, arginine, ascorbate, asparagine, aspartate, betaine, carnitine, choline, citrate, creatine, creatinine, dimethylamine (DMA), ethanol, formate, fumarate, glucose, glutamate, glutamine, glycerol, glycine, histidine, isoleucine, lactate, leucine, lysine, malate, mannose, methanol, methionine, N,N-dimethylglycine (DMG), acetylcarnitine, ornithine, phenylalanine, proline, pyruvate, serine, serotonin, succinate, taurine, threonine, trimethylamine (TMA), tryptophan, tyrosine, uracil, urea, uridine and valine. Samples were corrected for dilution by multiplying by a correction factor calculated by the ratio of the final sample volume divided by the initial volume of serum. Those samples for which there was limited volume were excluded from NMR analysis.

### Statistics

Unless otherwise indicated, all comparisons between the Fosamax group and either the sham or OVX group regarding bone measurements were made using Student's *t*-test, and comparison between multiple groups were made using ANOVA followed by Bonferroni's multiple *t* test. For metabolite data, all comparisons were made using Kruskal-Wallis non-parametric analysis followed by Dunn's multiple comparison test. For time-course data (body weight), analysis was accomplished using repeated measures two-way ANOVA. Statistical significance was defined as *p*<0.05. All data are expressed as mean ± standard error (SEM) or mean ± standard deviation (SD). Data sets were analyzed for statistical significance using Prism GraphPad 6 software (GraphPad Software Inc., San Diego, CA). Multivariate data analysis (Principal Component Analysis (PCA) and Partial Least Squares-Discriminant Analysis (PLS-DA)) was performed on log_10_ - transformed metabolite concentrations using SIMCA-P (version 13.0, Umetrics, Umeå, Sweden) as described in Slupsky [24]. The number of components was chosen using a 7-fold cross validation rule. The quality of the models were judged by the goodness-of-fit parameter (R^2^X), and the predictive ability parameter (Q^2^), as well as permutation testing (for the PLS-DA model), where each data point was randomly assigned a class 100 times, and the corresponding R^2^ and Q^2^ calculated and compared with the original model.

## Results

### Total body weight

**Figure 1:**
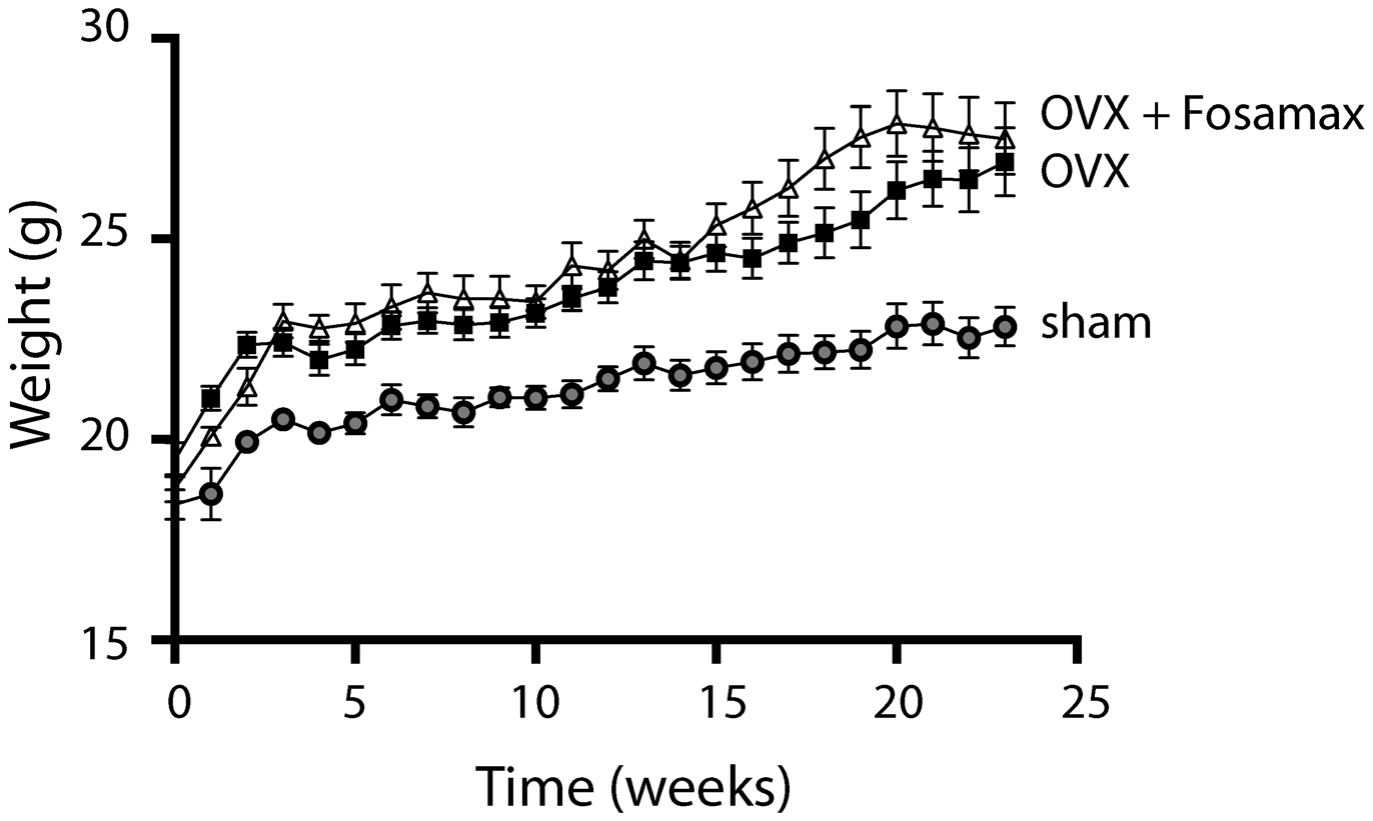
Body weight of mice over 23 weeks. Body weight of sham (circle), OVX (square), and OVX + Fosamax (triangle) C57BL/6 mice over 23 weeks. Time 0 represents the time of surgery. Analyzed by 2-way analysis of variance (ANOVA). Each value is expressed as mean ± SEM.

**Figure 2:**
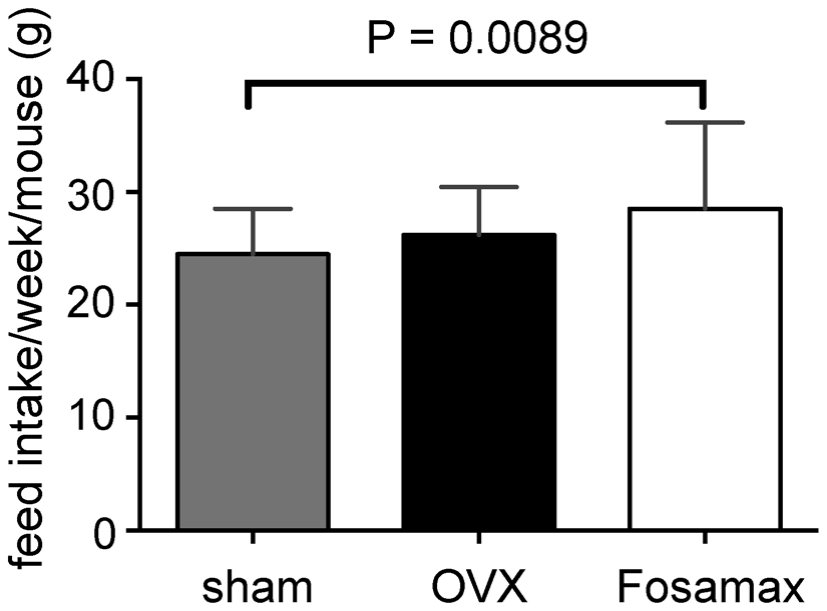
Weekly food intake. Average weekly food intake of sham C57BL/6 female mice compared with ovariectomized mice with and without Fosamax treatment during weeks 8–23. Analyzed by analysis of variance (ANOVA) followed by Bonferroni's multiple *t* test. Statistical significance is defined as *p*<0.05. Each value is expressed as mean ± SD.

Average body weight for each group of mice over 23 weeks after surgery is shown in Figure 1. Analysis by repeated measures 2-way ANOVA revealed a significant effect of time (p<0.0001) and treatment (p = 0.0011) on each group, and a significant interaction effect (p<0.0001). Calculation of the % increase in body weights of each of the groups at week 22 revealed an increase of 23% (sham), 36% (OVX) and 47% (Fosamax), compared with the starting weight. Analysis of the rate of weight gain over 23 weeks revealed significantly different slopes (*p*<0.0001), with sham mice gaining: 0.15±0.01 g/w (R^2^ = 0.48); OVX mice gaining: 0.24±0.02 g/w (R^2^ = 0.56); and Fosamax mice gaining: 0.32±0.02 g/w (R^2^ = 0.56). Interestingly, average food intake per week per mouse from week 8 until the end of the experiment revealed OVX and sham mice had similar food intakes, Fosamax and OVX had similar food intakes, but Fosamax mice had significantly higher food intake compared with sham mice (Figure 2).

### Micro-computed tomography analysis

**Figure 3:**
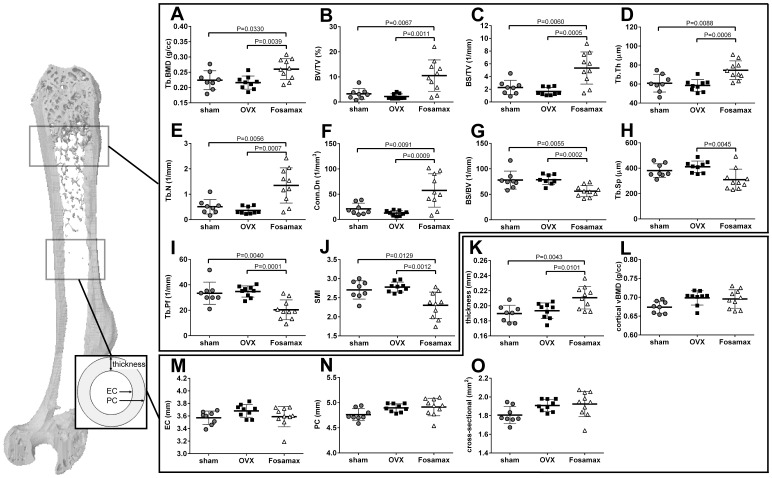
Micro-CT femur parameters. Bone characteristics of sham C57BL/6 female mice compared with ovariectomized mice with and without Fosamax treatment for 23 weeks. (A-J) Bone parameters of the distal femur analyzed by micro-CT. (A) Tb.BMD, trabecular bone mineral density; (B) BV/TV, bone volume/tissue volume; (C) BS/TV, bone surface/tissue volume; (D) Tb.Th, trabecular thickness; (E) Tb.N, trabecular number; (F) Conn.Dn, connectivity density; (G) BS/BV, bone surface/bone volume; (H) Tb.Sp, trabecular separation; (I) Tb.Pf, trabecular pattern factor; (J) SMI, structure model index. (K-O) Cortical bone parameters of the middle diaphysis analyzed by micro-CT. (K) Thickness, cortical bone shell thickness; (L) cortical BMD, cortical bone mineral density; (M) EC, endosteal circumference; (N) PC, periosteal circumference; (O) cross-sectional, cross-sectional area. Analyzed by analysis of variance (ANOVA) followed by Bonferroni's multiple *t* test. Statistical significance is defined as *p*<0.05. Each value is expressed as mean ± SD.

**Figure 4:**
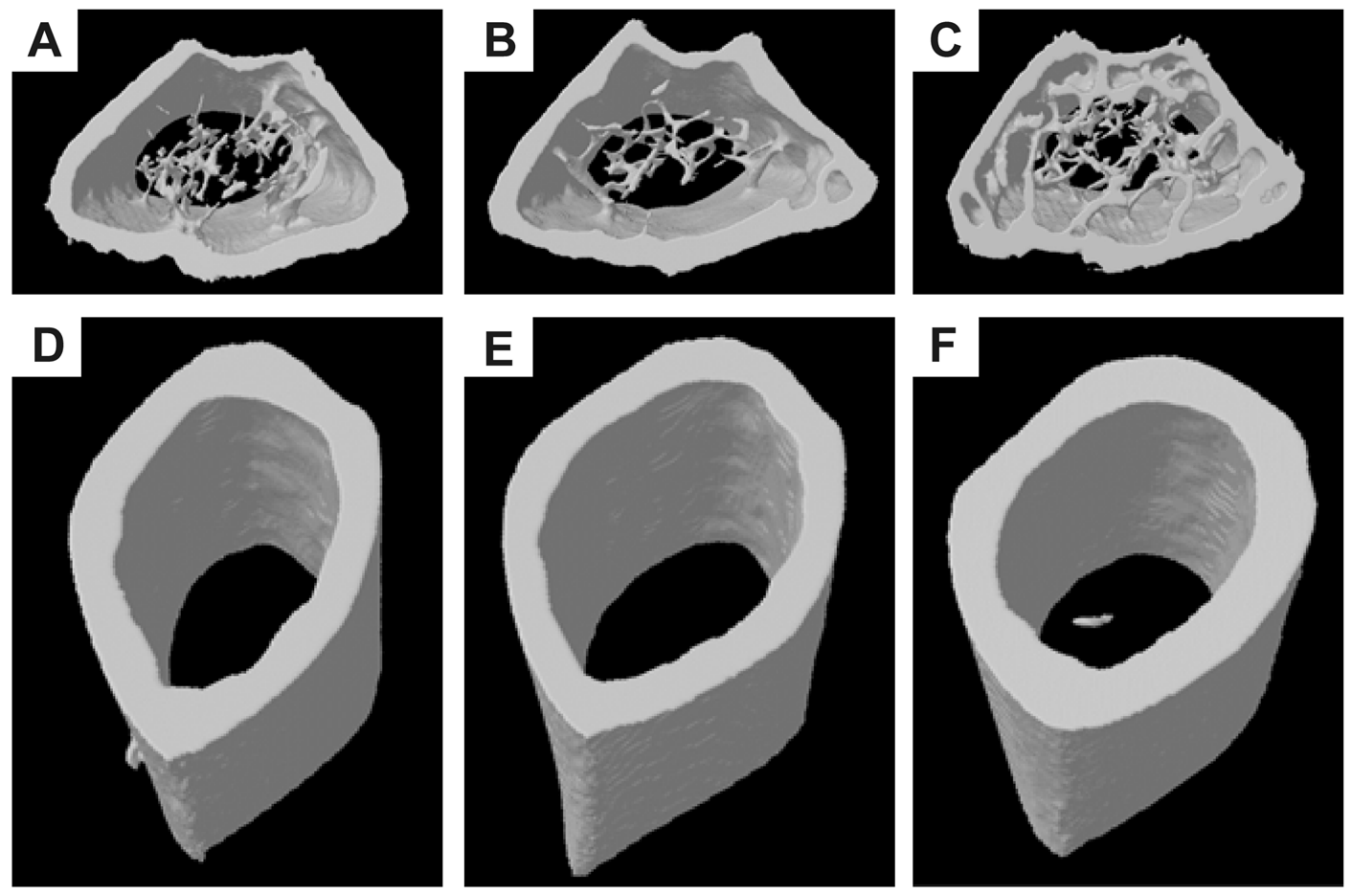
Micro-CT three-dimensional images. Femur images of sham, OVX and OVX + Fosamax C57BL/6 female mice treatment for 23 weeks. The distal femur region of: (A) sham mice, (B) OVX mice, and (C) Fosamax-treated mice. Femoral mid-diaphysis of: (D) sham mice (E) OVX mice, and (F) Fosamax-treated mice.

After 23 weeks of treatment, the trabecular bone parameters in the distal femur were analyzed by micro-CT and are shown in Figure 3. No significant difference was observed between sham and OVX mice for any of the parameters; however, Tb.BMD, BV/TV, BS/TV, Tb.Th, Tb.N and Conn.Dn were significantly higher, and BS/BV, Tb.Sp, Tb.Pf, and SMI were significantly lower in Fosamax-treated mice compared with OVX mice. Measurement of the cortical bone in the middle of the femur revealed that Fosamax-treated mice had significantly thicker cortical bone compared with sham and OVX mice suggesting increased bone strength. However, Fosamax-treated mice did not show significantly different cortical BMD, EC, PC and cross-sectional area compared with OVX mice. Three-dimensional (3D) micro-CT images of the middle diaphysis of the distal femur with trabecula are shown in Figure 4. A larger trabecular network was observed in the Fosamax treated mice compared with sham or OVX mice.

### Serum osteoblast and osteoclast activity, and calcium and phosphate concentrations

**Figure 5:**
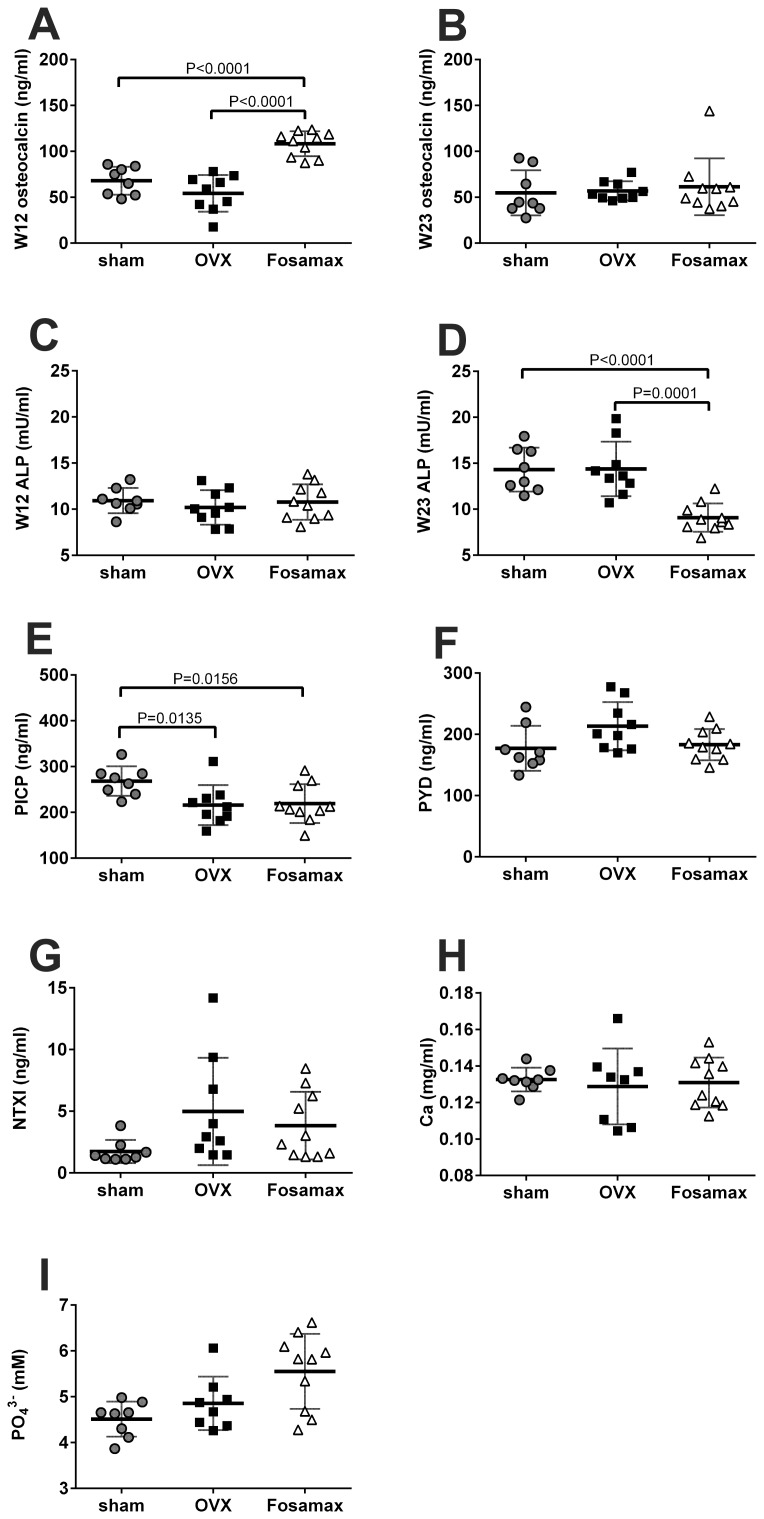
The concentrations of osteoblast and osteoclast indicators, calcium and phosphate in serum. Effects on serum osteoblast and osteoclast indicators, calcium and phosphate concentration of sham C57BL/6 female mice compared with ovariectomized mice with and without Fosamax treatment for 12 (W12) or 23 (W23) weeks. (A-E) Osteoblast indicators. (A) W12 osteocalcin; (B) W23 osteocalcin; (C) W12 ALP, alkaline phosphatase; (D) W23 ALP; (E) PICP, procollagen I C-terminal propeptide after 23 weeks treatment. (F.G) Osteoclast indicators. (F) PYD, pyridinoline after 23 weeks treatment. (G) NTXI, cross-linked N-telopeptide of type I collagen after 23 weeks treatment. Serum concentration of: (H) calcium after 23 weeks treatment, and (I) phosphate after 23 weeks treatment. Analyzed by analysis of variance (ANOVA) followed by Bonferroni's multiple *t* test. Statistical significance is defined as *p*<0.05. Each value is expressed as mean ± SD.

Activity of osteoblasts and osteoclasts were measured indirectly through a series of markers (Figure 5). For osteoblasts, elevated concentrations of osteocalcin, ALP, and PICP indicate elevated osteoblast activity, whereas elevated osteoclast activity is indicated through higher levels of PYD and NTXI. The serum osteocalcin concentration of Fosamax-treated mice was significantly higher than sham and OVX at week 12, but no difference was observed at week 23. Serum ALP showed no significant difference among three treatments at week 12, but Fosamax-treated mice had significantly lower levels than sham and OVX at week 23. Sham mice showed higher PICP levels than Fosamax and OVX at week 23. These results suggest little negative effect of Fosamax or ovariectomy on the indicators of osteoblast activity. In terms of the indicators of osteoclast activity, no significant difference between Fosamax treated mice and sham or OVX mice was observed. Sham mice showed lower PYD and NTXI than OVX, but the difference was not considered significant (*p* = 0.070 and 0.057, respectively) (Figure 5). Additionally, Fosamax treatment did not affect the serum concentration of calcium or phosphate significantly (Figure 5).

### Serum Metabolomics

**Figure 6:**
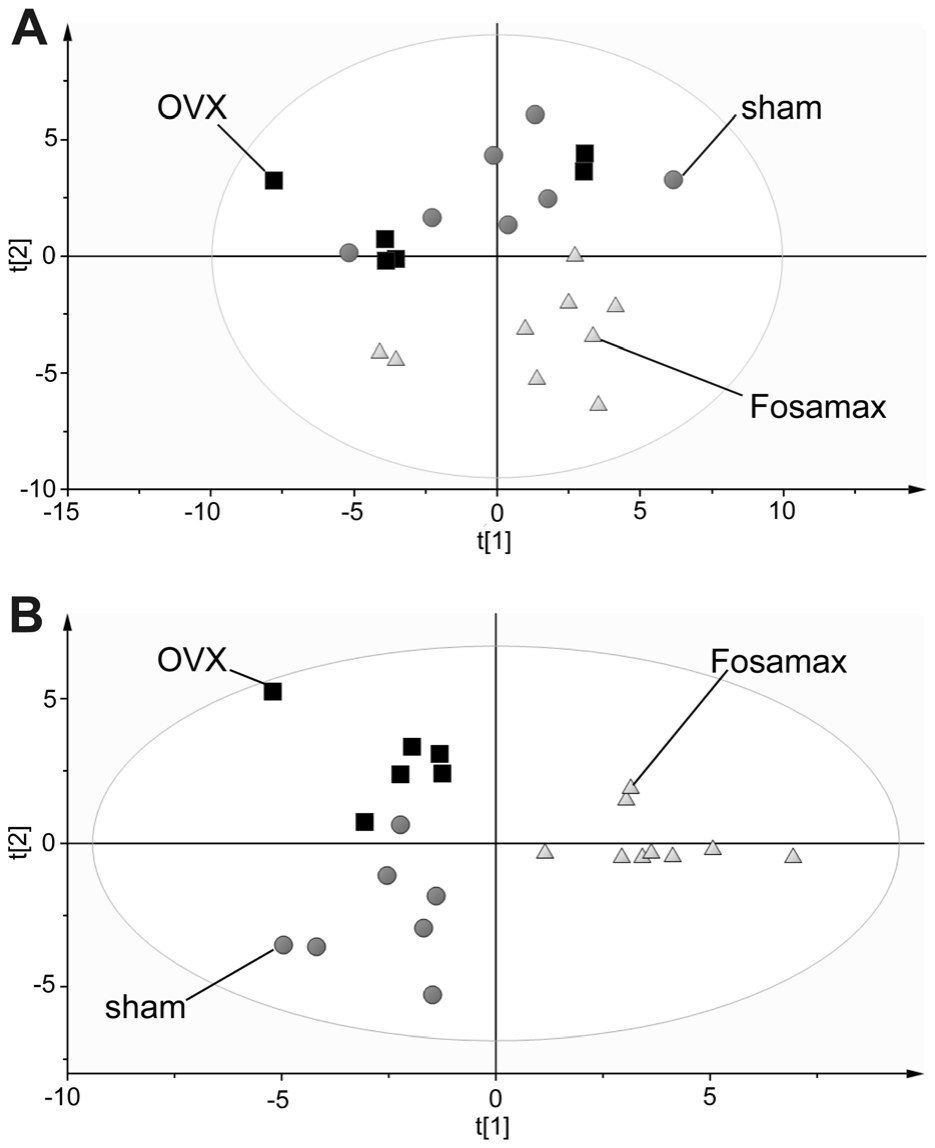
Comparison of metabolic composition of serum. (A) PCA (R^2^X = 0.642, Q^2^ = 0.178) and (B) PLS-DA (R^2^Y = 0.915, Q^2^ = 0.700) models of serum metabolite concentrations of sham C57BL/6 female mice compared with ovariectomized mice with and without Fosamax treatment for 23 weeks. Permutation testing revealed that the model was valid, with all values of the permuted R^2^Y and Q^2^ data being significantly less than the original model (p<0.001).

PCA of metabolite concentrations obtained from the analysis of mouse serum after 23 weeks is shown in Figure 6A. Fosamax-treated mice were observed to have a completely different serum metabolite profile in comparison with the OVX and sham mice. To maximize the separation between sham, OVX, and Fosamax, PLS-DA was employed and is shown in Figure 6B. The largest difference was between the Fosamax-treated mice and the sham or OVX mice as shown by separation in the first component, whereas a smaller difference between sham and OVX mice was observed in the second component. Twelve metabolites were primarily responsible for the difference between OVX and Fosamax-treated mice after 23 weeks of treatment, and are summarized in Table 1. TCA-cycle intermediates (citrate, 2-oxoglutarate, malate, fumarate, and succinate), amino acids (taurine), glucose, 3-hydroxybutyrate (a ketone), and others (acetate, allantoin, and ethanol) were significantly higher in Fosamax-treated mice compared with OVX, while aspartate was significantly lower.

**Table 1:** Comparison of serum metabolite concentrations after 23 weeks.

Metabolite^a^ (µM)		Sham	OVX	Fosamax	*p*-value	Adjusted *p*-value OVX vs Fosamax	Adjusted *p*-value Sham vs OVX
TCA cycle	Citrate	293.1±24.6	263.5±38.2	365.2±37.6	<0.0001	0.0020	>0.99
	2-Oxoglutarate	32.3±12.0	35.3±2.7	49.7±9.8	0.0010	0.0202	>0.99
	Malate	106.4±26.6	93.0±38.4	176.4±47.4	0.0010	0.0055	>0.99
	Fumarate	10.9±5.0	13.0±5.0	19.6±5.1	0.0067	0.0693	>0.99
	Succinate	436.8±139.9	389.7±138.3	679.8±187.3	0.0165	0.0341	>0.99
Amino acids	Aspartate	39.1±10.3	38.2±9.0	23.8±7.2	0.0020	0.0325	>0.99
	Taurine	460.8±95.1	467.6±116.0	669.2±127.6	0.0040	0.0341	>0.99
	Glycine	313.8±41.1	249.8±42.5	289.5±38.5	0.0482	0.5338	0.0468
Energy metabolism	Glucose	13188.6±2517.5	14309.8±1897.3	18547.8±2747.4	0.0007	0.0468	>0.99
Ketones	3-Hydroxybutyrate	110.8±44.8	76.2±17.9	168.5±63.8	0.0052	0.0081	0.6103
Others	Acetate	65.1±19.2	44.6±16.1	78.7±14.9	0.0095	0.0110	0.2787
	Allantoin	165.6±50.3	100.4±12.2	138.7±32.8	0.0047	0.0609	0.0089
	Ethanol	188.4±81.4	85.1±35.5	300.1±233.9	0.0025	0.0062	0.0397
	Dimethylamine	4.1±1.5	1.9±0.7	2.7±0.6	0.0015	0.4062	0.0012

a Significantly different metabolites between OVX and Fosamax-treated mice or Sham and OVX mice. Each value is expressed as mean ± SD. *P*-values were calculated using the Kruskal-Wallis test, and adjusted *p*-values were calculated using Dunn's multiple comparison test. Alpha was set to 0.05.

Comparison of sham and OVX mice after 23 weeks revealed four metabolites that were significantly different (Table 1). Glycine, DMA, ethanol and allantoin were all significantly higher in sham mice compared with OVX mice.

## Discussion

Type 1 (postmenopausal) osteoporosis is characterized by hormonal deprivation resulting in accelerated bone turnover and bone loss [25]. The OVX rat and mouse are classic models used to study postmenopausal bone loss since removal of the ovaries mimics postmenopausal hormonal changes that occur in humans, including decreases in estrogen and progesterone [26]. In this study, OVX mice were compared with OVX mice treated with Fosamax, as well as mice that received sham surgery. Interestingly, weight gain of OVX mice over 23 weeks was higher than sham mice, even though their food intake was similar. It has previously been shown that estrogen deficiency can cause higher body weight and body fat deposition [27], [28], which may partially explain our results.

Metabolomics can measure changes in the levels of metabolites found in biological fluids and tissues, which can produce a quantitative and comprehensive indication of metabolic pathway activity. It can offer information to understand the effect of diet, drugs and disease, and assessment of a disease state [29], [30]. Few metabolic differences between OVX and sham mice were observed. One interesting difference was the significantly lower glycine concentration in OVX mice compared with sham. Glycine has been shown to be inversely associated with cholesterol, phospholipid, free fatty acid, and triglyceride accumulation [31], which may indicate changes in fat metabolism. Lower dimethylamine, and ethanol may be related to changes in gut microbial metabolism, and lower allantoin may indicate lower oxidative stress [32] in mice with ovariectomy compared with sham.

Despite the minor metabolic changes that occurred in the OVX mice, they were similar to sham mice with respect to bone-related measurements. We speculate that the age of the mice may have been a factor. Indeed, a report evaluating age-related changes in trabecular architecture in C57BL/6J mice showed that the trabecular bone volume (BV/TV) is greatest at 6–8 weeks of age, and declines steadily thereafter. Moreover, trabecular bone loss, as measured by trabecular number (Tb.N), is most rapid between 2–6 months of age, with a more gradual decline thereafter [33]. Our study commenced when the mice were 3 months of age (12 weeks), and continued until the mice were ∼9 months of age (35 weeks).

Fosamax-treated ovariectomized mice revealed increased Tb.BMD, BV/TV, Tb.Th, Tb.N, and cortical bone thickness as well as decreased Tb.Sp, and SMI compared with both OVX and sham, which demonstrates that Fosamax appears to increase bone mass and strength as has been previously reported [34]–[36]. Furthermore, some reports indicate that bisphosphonates suppress the resorption and formation of bones, thereby decreasing bone turnover and lowering bone loss [37], [38]. Several serum indicators of osteoblast and osteoclast activity, indicators of bone turnover, were examined. Indicators of osteoblast activity included ALP, osteocalcin, and PICP. Fosamax-treated mice exhibited lower ALP, but no significant difference in osteocalcin or PICP at week 23 compared with the OVX mice. Various studies have shown that alendronate treatment either decreases serum osteocalcin significantly, or brings about no significant change in osteoblast activity [34], [37], [39], which supports the observations presented here. Interestingly, osteocalcin levels in Fosamax-treated mice at week 12 were higher than sham or OVX mice, and higher than all three groups of mice at 23 weeks. We speculate that the cholecalciferol found in Fosamax may be responsible for the initial increase of osteocalcin observed at 12 weeks [40]. No significant difference in either of the osteoclast activity indicators (PYD and NTXI) after Fosamax treatment for 12 or 23 weeks was observed.

Administration of Fosamax for 23 weeks to OVX mice resulted in a significantly higher rate of weight gain over the course of the experiment compared with both sham and OVX mice. While the OVX mice receiving Fosamax treatment had a slightly elevated intake of chow compared with sham, intake was not significantly different between OVX and Fosamax-treated mice, suggesting that other factors may be responsible for the increased rate of weight gain of Fosamax mice. Interestingly, other studies in rats have shown that alendronate treatment results in higher body weight compared with OVX [28]. One possibility of the increased weight gain could be the neo-formation of bone tissue [41]. However, other factors may also affect body weight, including changes in global metabolism through changes in adiponectin, leptin and energy metabolism (glucose, 3-hydroxybutyrate and taurine), as well as activity of osteoblast cells. Interestingly, adiponectin and leptin, the main circulating peptides secreted by adipose tissue, have been shown to be involved with regulation of bone metabolism [42], [43], and treatment with alendronate for 12 months has been shown to slightly decrease serum leptin [44].

One interesting observation was a decrease in osteocalcin at week 23 compared with week 12 in Fosamax-treated mice. Osteoblasts are known to secrete hormones that affect energy metabolism. Indeed, mice deficient in osteocalcin are obese, having decreased β-cell proliferation and secretion, greater insulin resistance, and decreased energy expenditure [45]–[47]. Insulin is an important molecule associated with bone remodeling and energy metabolism [48]. While insulin was not directly measured in this study, serum metabolites of Fosamax-treated mice were compared with OVX mice, revealing higher serum glucose and higher 3-hydroxybutyrate in Fosamax-treated mice compared with OVX mice. Hyperglycemia has been correlated with diabetic ketoacidosis, which can result in stimulation of lipolytic pathways, resulting in free fatty acids and ketone bodies in the blood [49].

However, it is puzzling that there is an almost 2-fold higher concentration of osteocalcin in Fosamax-treated mice at week 12, which then appears to return to levels similar to OVX and sham mice at week 23. It is tempting to speculate that this change in osteocalcin levels may lead to insulin resistance and lipolysis [48], followed by increased weight gain and risk of diabetes [39], [45], [47]. Indeed, most of the increased weight gain of the Fosamax treated mice was in the later part of the experiment, and these results could suggest that prolonged use of the drug may have negative consequences. However, this will need to be elucidated in a further study. Indeed, there is much controversy in the literature on the correlation of bisphosphonate therapy with glycemia, diabetes and body weight [50]–[52], and whether these observations are restricted to mice or may be extended to humans remains to be clarified.

Other metabolites that may be connected with osteoporosis include 3-hydroxybutyrate and taurine [53]-[56]. 3-hydroxybutyrate has been shown to reduce bone absorption, maintain normal bone function and femur bone mineral density, thus reducing osteoporosis [53]. We observed higher 3-hydroxybutyrate in Fosamax-treated mice. Our results also showed taurine was higher in Fosamax-treated mice. Interestingly, taurine supplementation has been shown to increase bone mineral density in rats [54], [55]. It can bind divalent cations, including Mg^2+^, and has an important role in bone remodeling and skeletal development [56].

Our results showed that while Fosamax increased bone mineral density and lowered bone loss, it only decreased serum osteoblast activity slightly, and did not affect osteoclast activity. Serum metabolomics revealed that Fosamax primarily affected energy metabolism. Specifically, Fosamax increased the concentrations of serum glucose, 3-hydroxybutyrate and taurine, which have been shown to be positivity related to bone health, but also significantly increased the concentrations of TCA cycle intermediates, suggesting that mitochondrial function may also be affected by Fosamax treatment. Significantly increased weight gain of Fosamax-treated mice, particularly in the later weeks of treatment, suggest that long-term Fosamax use appears to impact metabolism, but it is unclear at this stage how these metabolic changes may lead to complications observed with long-term use of bisphosphonates. Future studies will explore changes in serum insulin, adiponectin, and leptin with treatment, as well as determine global metabolic changes at 12 weeks to compare with 24 weeks to determine the length of time of maximum benefit.
